# End-to-End Automated Lane-Change Maneuvering Considering Driving Style Using a Deep Deterministic Policy Gradient Algorithm

**DOI:** 10.3390/s20185443

**Published:** 2020-09-22

**Authors:** Hongyu Hu, Ziyang Lu, Qi Wang, Chengyuan Zheng

**Affiliations:** 1 State Key Laboratory of Automotive Simulation and Control, Jilin University, Changchun 130022, China; l992149335@163.com (Z.L.); wqi19@mails.jlu.edu.cn (Q.W.); 2 Design School, Loughbrough University, London E20 3BS, UK; c.zheng-19@student.lboro.ac.uk

**Keywords:** intelligent vehicle, automated lane change, driving style, reinforcement learning, deep deterministic policy gradient

## Abstract

Changing lanes while driving requires coordinating the lateral and longitudinal controls of a vehicle, considering its running state and the surrounding environment. Although the existing rule-based automated lane-changing method is simple, it is unsuitable for unpredictable scenarios encountered in practice. Therefore, using a deep deterministic policy gradient (DDPG) algorithm, we propose an end-to-end method for automated lane changing based on lidar data. The distance state information of the lane boundary and the surrounding vehicles obtained by the agent in a simulation environment is denoted as the state space for an automated lane-change problem based on reinforcement learning. The steering wheel angle and longitudinal acceleration are used as the action space, and both the state and action spaces are continuous. In terms of the reward function, avoiding collision and setting different expected lane-changing distances that represent different driving styles are considered for security, and the angular velocity of the steering wheel and jerk are considered for comfort. The minimum speed limit for lane changing and the control of the agent for a quick lane change are considered for efficiency. For a one-way two-lane road, a visual simulation environment scene is constructed using Pyglet. By comparing the lane-changing process tracks of two driving styles in a simplified traffic flow scene, we study the influence of driving style on the lane-changing process and lane-changing time. Through the training and adjustment of the combined lateral and longitudinal control of autonomous vehicles with different driving styles in complex traffic scenes, the vehicles could complete a series of driving tasks while considering driving-style differences. The experimental results show that autonomous vehicles can reflect the differences in the driving styles at the time of lane change at the same speed. Under the combined lateral and longitudinal control, the autonomous vehicles exhibit good robustness to different speeds and traffic density in different road sections. Thus, autonomous vehicles trained using the proposed method can learn an automated lane-changing policy while considering safety, comfort, and efficiency.

## 1. Introduction

In recent years, with the continuous growth in car ownership, issues such as traffic safety and traffic congestion have become increasingly serious. Frequent road traffic accidents lead to casualties and property losses, whereas traffic congestion adds additional pressure to urban traffic systems. According to statistical results, among the key causes of traffic accidents, such as drivers, vehicles, and environmental factors, accidents due to drivers alone are as high as 94% ± 2.2%. Here, accidents due to cognitive errors, decision-making mistakes, and improper operation account for 41%, 33%, and 11% of traffic accidents, respectively [1]. Therefore, in the current context, an intelligent vehicle that can replace human drivers and realize automated driving has emerged as a key research subject for the future development of the entire automobile industry.

In an actual driving process, a driver exhibits three basic driving behaviors: car-following, lane-changing, and free driving [2]. Among them, the lane-changing process requires the driver to pay attention to the vehicles moving in the current lane and in the target lane simultaneously, in order to make decisions and implement comprehensive lateral and longitudinal control. This makes the lane-changing decision more complex than other driving behaviors. In addition, realizing anthropomorphic decision-making in the process of automated driving has been challenging. Current research on intelligent vehicle decision algorithms is mainly based on the design method of rules [3,4]. This type of method establishes behavior rules related to the driving process through an advanced “expert system” and tests, and then verifies them in specific scenarios. However, when vehicles are in complex scenarios and driving conditions, such as sudden accidents, this type of method cannot achieve satisfactory results [5]. Therefore, to deal with more complex scenarios, a decision-making method for intelligent vehicles based on reinforcement learning is a major milestone. This method uses a self-learning intelligent control algorithm that enables the agent to constantly interact with the environment. It helps intelligent vehicles to cope with complex scenarios independently. Reinforcement learning is a typical experience-driven and self-learning method, enabling an agent to find an optimal strategy to complete tasks through continuous “trial and error” and feedback learning while interacting with the environment. It has been used to solve practical engineering problems that can be modeled as a Markov decision process [6]. Moreover, deep reinforcement learning combines the perception ability of deep learning with the decision-making ability of reinforcement learning, thus bringing complementary advantages and providing a new solution for perception and decision-making in complex scenarios. Thus, deep reinforcement learning has emerged as another feasible scheme for automated lane changing and automatic driving of intelligent vehicles [7].

A lane-changing operation is a typical time sequential problem, in which the completion of a task requires a series of actions, and the execution of the current action will affect whether the objective of the task can be accomplished (e.g., changing lane successfully). Reinforcement learning is suitable for solving this type of problem [8].

The main research objective of this study was to design a deep deterministic policy gradient (DDPG) algorithm to control the lane-changing behavior of autonomous vehicles, and to characterize the aggressive and conservative driving styles by considering different expected lane-changing distances when designing the reward function. Moreover, a simplified traffic flow scene was constructed to verify the difference between the lane-changing process and the lane-changing time under different driving styles. Complex traffic flow scenes were constructed to verify whether autonomous vehicles under the decision control of the algorithm can complete a series of driving tasks (such as lane keeping and autonomous lane-changing), and the differences were compared in terms of the reward, collision rate, and success rate in the lane-changing process under different driving styles. Finally, we verified whether the agent under the control of the algorithm can ensure security, efficiency, and comfort while changing lanes.

The rest of this article is organized as follows. Section 2 summarizes the related research. Section 3 introduces the implementation process of the DDPG algorithm and related MDP design. In Section 4, the software and hardware facilities of the experiment are explained and related problems are studied by designing two different traffic scenes. Section 5 gives the corresponding training and verification results of the two traffic scenes. Section 6 gives a summary of the overall research and presents directions for future work.

## 2. Related Works

The conventional method for autonomous lane changing is based on predefined rules and clearly designed models. Most of these methods introduce virtual lane tracks (i.e., the reference tracks planned by the path) or a series of track points in order to follow the tracks in the process of changing lanes. These methods are unsuitable for the unpredictable scenarios encountered in the process of actual driving.

In recent years, reinforcement learning has been developed and improved. The most basic reinforcement learning algorithm, namely the Q learning method, mainly uses a table to record the value function [9]. It is a temporal-difference learning method based on the value function: however, this method is generally applicable to a discrete action space. Once the problem becomes more complex, the table that records the value function will consume more space and slow down the algorithm, resulting in a “dimensional disaster” phenomenon. Kretchmar improved the reinforcement learning algorithm using a parallel learning technology, which improved the running speed of the algorithm [10]. Mnih et al. proposed a deep Q network (DQN) model by combining the conventional Q learning algorithm with a deep learning neural network [11], where the deep neural network is intuitively used to approximate the policy or value function. In 2015, DeepMind launched the method of double DQN [12], which further enriched the content of DQN. With increasing research, reinforcement learning has been derived from different research branches such as meta-learning [13,14] and inverse reinforcement learning [15,16].

With more advantages and increased popularity of reinforcement learning, some scholars have applied this method to the automatic driving control of vehicles. Zhang et al. constructed an environment model based on natural driving data [17]. The input to the model was composed of high-dimensional data (including road information obtained by video processing) and low-dimensional data obtained by sensors. The sensor data were fused to feed a neural network and determine the vehicle acceleration in the current state, including the acceleration, deceleration, and uniform speed. Fares and Gomaa balanced the demand and capacity of highways by controlling the number of vehicles entering the mainstream highway from a ramp merger area [18]. They defined the state space as a 3D space, and the action space was defined with two actions: red and green. Yu et al. applied a deep Q network that combines a deep neural network and Q learning to structured urban roads and studied the overtaking strategy for driverless buses [19]. The lane ID, the speed of the autonomous vehicle, the speeds of other vehicles, and the distance between the autonomous vehicle and other vehicles were used as the state space. The action space included the action of lane change and acceleration, lane change and uniform speed, lane change and deceleration, no lane change and acceleration, no lane change and uniform speed, and no lane change and deceleration. The agent was trained and verified in a simulation environment. Although the use of a discrete action space can help simplify the problem, it is not conducive to the optimal policy for solving practical problems. Sallab et al. compared lane-keeping task learning in the discrete action space and continuous action space [20]. The experimental results showed that both strategies can realize the task of lane keeping. Nevertheless, with the learning method of the continuous action space, the final steering action value is smoother, thus meeting a driver’s comfort requirements.

Scholars have studied the problem of continuous action space. For example, Wang and Chan analyzed the driving behavior of on-ramp vehicle confluence by reinforcement learning [21]. In this method, the action and state spaces are continuous, and the design of the reward function takes safety, comfort, and efficiency into account. Wang et al. applied the reinforcement learning algorithm to the study of automated lane-changing behavior of unmanned vehicles in an interactive driving environment [8]. Through the combination of a deep neural network and the Q-learning algorithm, a quadratic Q-function approximator was designed, and a vehicle control strategy with a continuous state space and action space was established. The longitudinal speed adapts to the current traffic flow state based on the intelligent driver model. Laterally, it relies on the reinforcement learning algorithm to make decisions depending on the current environmental state. In [22], Gu et al. proposed a fast adaptive learning method for supervised deep reinforcement learning and realized the path following of a self-driving vehicle with high-dimensional input. This method combines the conventional feedback control method with deep reinforcement learning and uses the improved algorithm to train on TORCS. A comparison showed that the improved algorithm is more effective than the simple DDPG algorithm, with a faster learning ability in the steering operation task. Liu et al. employed data obtained in a simulation environment and real driving data as the state input to the agent to train a neural network, update the network parameters by introducing supervisory loss, and make the agent learn as much as possible from the real data to improve the training process [23]. In [24], Wang et al. used continuous action space and the DDPG reinforcement learning algorithm to study the lateral control of vehicle lane-changing behavior. An intelligent driver model was used for the longitudinal control, considering the relative speed and distance between the agent and the vehicle in front, and finally, a suitable acceleration for following the vehicle in front was determined. For lateral control, reinforcement learning is used to determine the yaw velocity of the lane-changing process on the basis of the gap selection module. In the above research, although continuous state space and action space were used, the lane-changing problem was only divided into longitudinal or lateral control, i.e., reinforcement learning was only used to solve longitudinal or lateral problems.

Current related research on the perception layer has mostly been conducted by analyzing the images obtained using cameras, radar data, GPS, and CAN data, or using the relative position of the vehicle in the environment and other information to perceive the environment. Pan et al. used a two-stage automatic encoder–decoder for image translation, and then used the real image synthesized by the virtual image as the input to train a deep reinforcement learning agent [25]. In [26], Jaritz et al. carried out end-to-end control of an agent under race driving. The steering, throttle, foot brake, and handbrake were controlled using the image information obtained by the front camera, and the asynchronous advantage actor–critic (A3C) algorithm was used for training in the game World Rally Championship 6 (WRC6). In [27], An et al. considered the position coordinates and speeds of the agent and other vehicles as the input to the state space, for training. In their study on the following behavior of two vehicles, Zhu et al. selected the speed, relative distance, and relative speed of two vehicles in the state space [28]. In [20], Sallab et al. realized the perception of the environment through sensors such as camera, lidar, and millimeter wave radar, and the data were fused using multi-sensors to train the driving task of lane keeping in TORCS. Another study was conducted on road edge detection of structured roads based on lidar alone. In [29], Wang et al. proposed a method for road edge detection of structured roads based on robust 3D-lidar.

## 3. Problem Formulation

**Figure 1:**
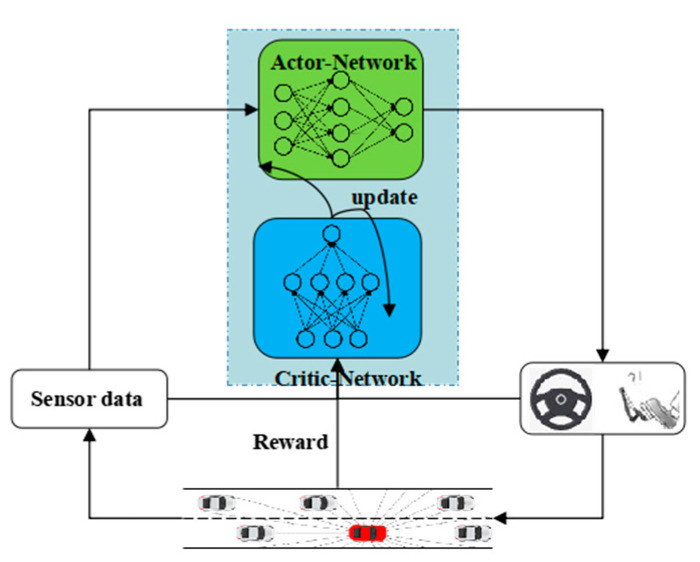
Framework of automated lane-changing based on the deep deterministic policy gradient (DDPG) algorithm.

In this study, the proposed end-to-end automated lane-changing method based on the DDPG algorithm completely depends on the data measured by single-line lidar sensor in the simulation environment. The data include the distance between the agent and the surrounding vehicles, and the road boundaries in all directions. A safe driving area in the agent lane-changing process is formed using these data. Both the state space and the action space are continuous, and the reward function considers the security, comfort, and efficiency. Figure 1 shows the automated lane-changing framework based on the proposed DDPG algorithm. In this section, we mainly introduce the methods used in the study to solve the problem of automated lane change. This includes the basic theory of reinforcement learning, DDPG algorithm, and the design of the state space, action space, reward function, and neural network structure for automated lane change.

### 3.1. Reinforcement Learning

**Figure 2:**
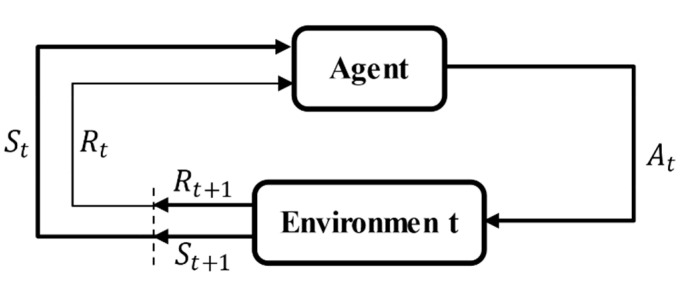
Basic framework of reinforcement learning.

Reinforcement learning is a method that can obtain an optimal policy through the continuous interaction between the agent and the environment and change in the action based on the feedback of the environment to the action. As shown in Figure 2, the agent takes its own information in the current environment as the state s∈S and generates an action a∈A based on the current state s. After performing the action, the agent gets the reward value R(s,a) and next state $s\in S^{\prime }$, and obtains the cumulative return through continuous iteration until an optimal policy $\pi^{*}$ is found [30].

### 3.2. DDPG Algorithm

The DQN algorithm has achieved success in Atari. However, it can only solve the problem of discrete action space. It cannot output the action-value function of each action for the continuous action space. Moreover, many control problems in real life often require the action space to be continuous. If the continuous action space is simply divided into many nearly continuous, discontinuous spaces, the rapid increase in the action dimensions will make it difficult to train the neural network to converge. Therefore, based on the DQN algorithm, the DDPG algorithm not only uses experience replay and independent target network methods, but also introduces the advantages of the DQN algorithm directly in the field of the continuous action space. The DDPG algorithm is detailed as follows:
**Algorithm 1** DDPG algorithmRandomly initialize critic network $Q(s,a|\theta^{Q})$ and actor $\mu \left(s|\theta^{\mu }\right)$ with weights $\theta^{\theta }$ and $\theta^{\mu }$ Initialize target network $Q^{\prime }$ and $\mu^{\prime }$ with weights $\theta^{Q^{\prime }}\leftarrow \theta^{Q}$, $\theta^{\mu^{\prime }}\leftarrow \theta^{\mu }$ Initialize replay buffer R **for** episode = 1, M **do**   Initialize a random process N for action exploration   Receive initial observation state $s_{1}$   **for** t = 1, T **do**     Select action $a_{t}=\mu \left(s_{t}|\theta^{\mu }\right)+$ Nt     according to the current policy and exploration noise     Execute action $a_{t}$ and observe reward $r_{t}$ and observe new state $s_{t+1}$     Store transition $<s_{t},a_{t},r_{t},s_{t+1}>$ in R     Sample a random minibatch of N transitions $<s_{i},a_{i},r_{i},s_{i+1}>$ from R     Set $y_{i}=r_{i}+\gamma Q^{\prime }(s_{i+1},\mu^{\prime }(s_{i+1}|\theta^{\mu^{\prime }})|\theta^{Q^{\prime }})$     Update critic by minimizing the loss: $L=\frac{1}{N}\sum_{i}\left(y_{i}-Q{\left(s_{i},a_{i}|\theta^{Q}\right)}^{2}\right)$     Update the actor policy using the sampled gradient:         $\nabla_{\theta_{o_{\mu }}}\mu {|}_{s_{i}}\approx \frac{1}{N}\sum_{i}\nabla_{a}Q\left(s,a|\theta^{Q}\right){|}_{s=s_{i},a=\mu \left(s_{i}\right)}\nabla_{\theta^{\mu }}\mu \left({s|\theta }^{\mu }\right){|}_{s=s_{i}}$
    Update the target networks:                 $\theta^{Q^{\prime }}\leftarrow \tau \theta^{Q}+\left(1-\tau \right)\theta^{Q^{\prime }}$                 $\theta^{\mu^{\prime }}\leftarrow \tau \theta^{\mu }+\left(1-\tau \right)\theta^{\mu^{\prime }}$ **end for** **end for**

**Figure 3:**
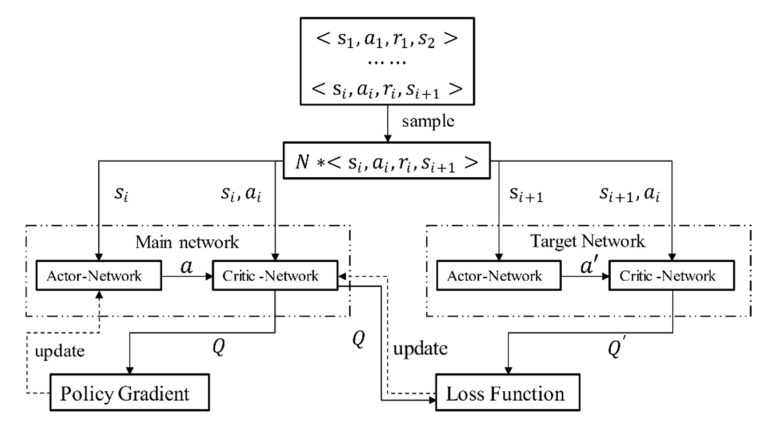
Neural network framework.

The DDPG algorithm [31] adopts the framework of the actor–critic algorithm, and adds the idea of the experience replay and random sampling. The process of establishing the independent target network in the DQN is also considered. It is developed on the basis of the DPG, so the algorithm can be considered as an integration of the Actor–Critic, DQN, and DPG. The Actor–Critic algorithm framework integrates the theory of policy search and value function approximation and is divided into two parts: actor-network and critic-network. The DDPG consists of two main networks and two target networks, as shown in Figure 3.

The input to the actor-network in the main network is the current state s, and the output is the action a selected depending on the policy $\mu (s_{t}|\theta^{\mu })$. The input to the critic-network in the main network is the current state s and the actual output action a, and the output is the calculated current Q value, i.e., $Q(s,a|\theta^{Q})$. This Q value updates the weight of the actor-network in the main network through the policy gradient, and is also used to calculate the loss function. The update function of the actor-network parameters in the main network is as follows:

(1) $$\nabla_{\theta_{\mu }}\mu {|}_{s_{t}}\approx \frac{1}{N}\sum_{t}\nabla_{a}Q\left(s,a|\theta^{Q}\right){|}_{s=s_{t},a=\mu \left(s_{t}\right)}\nabla_{\theta^{\mu }}\mu \left(s|\theta^{\mu }\right){|}_{s=s_{t}}$$

where N is the number of randomly sampled samples, $Q(s,a|\theta^{Q})$ is the action-value function, and $\mu (s|\theta^{\mu })$ is the deterministic policy.

The input to the actor-network in the target network is the next state $s^{\prime }$, and the output is the action $a^{\prime }$ selected based on the policy $\mu^{\prime }(s_{t+1}|\theta^{\mu^{\prime }})$. The input to the critic-network in the target network is the next state $s^{\prime }$ and the action $a^{\prime }$ output through the actor-network. The output is the corresponding $Q^{\prime }$ value of the target network, namely $Q\prime (s_{t+1},\mu \prime (s_{t+1}|\theta^{\mu \prime })|\theta^{Q\prime })$. By calculating the loss function between this Q′ value and the Q value calculated in the main network, the algorithm can update the weight of the critic-network in the main network by minimizing the loss function.

(2) $$y_{t}=r\left(s_{t},a_{t}\right)+\gamma Q\prime (s_{t+1},\mu (s_{t+1}|\theta^{\mu^{\prime }})|\theta^{Q^{\prime }})$$

(3) $$L=1/N*\sum_{i=t}(y_{t}-Q{\left(s_{t},a_{t}|\theta^{Q}\right)}^{2})$$

Functions (2) and (3) are the calculation formulae for the reward value and loss function, respectively, where $y_{t}$ is the current real reward value, $r\left(s_{t},a_{t}\right)$ is the current reward value, γ is the discount factor of the future reward value, $Q\prime (s_{t+1},\mu (s_{t+1}|\theta^{\mu^{\prime }})|\theta^{Q^{\prime }})$ is the action-state value function corresponding to the next moment, L is the loss function, and $Q(s_{t},a_{t}|\theta^{Q})$ is the action-value function of the current moment.

The independent target network method involves using the main network for single-step learning and iterative updating, and then assigning the parameters of the main network to the independent target network after a certain number of iterations. Unlike the method used in the DQN to assign the main network parameters to the target network at a certain number of steps, the DDPG uses a small number of updates to the parameters of the target network at each step. After a small batch of data is trained with a capacity of N, the soft update algorithm is used to update the parameters of the target network:

(4) $$\theta^{Q^{\prime }}\leftarrow \tau \theta^{Q}+\left(1-\tau \right)\theta^{Q^{\prime }}$$

(5) $$\theta^{\mu^{\prime }}\leftarrow \tau \theta^{\mu }+\left(1-\tau \right)\theta^{\mu^{\prime }}$$

### 3.3. Actor–Critic Network Structure Design

The DDPG algorithm used in this study depends on the typical actor–critic algorithm framework, which comprises two neural networks: an actor-network and a critic-network. Compared with the conventional reinforcement learning algorithm, the focus of the DDPG algorithm is more on the design and optimization of the actor–critic network structure. Considering that the real-time detection data from the single-line lidar are used as the original input to the deep reinforcement learning to solve the lane-changing problem of autonomous vehicles, the lidar data are low-dimensional unlike the image data collected by the camera. The essence of reinforcement learning in solving the automated lane-changing problem lies in solving the optimal vehicle lane-changing track. The DDPG algorithm uses a deep learning neural network to express the trajectory equation, and through the method of reinforcement learning, the decision-making of the agent is updated in the direction of maximizing the value of the reward function. In theory, a neural network with only one hidden layer is sufficient to describe any function: therefore, we select a fully connected neural network as the basic network structure of the actor-network and critic-network.

The original measurement data cannot be directly used as input in the training of neural networks. In general, to eliminate the dimensions and data differences between different data, the original data must go through pre-processing. Therefore, the data are uniformly mapped to the interval of [0, 1] using min–max normalization. The actor-network takes the preprocessed data as the input, connects with the fully connected layer, and uses the nonlinear tanh function as the activation function. The final output of the actor-network represents the steering, acceleration, and deceleration of the agent. The final output range of the steering wheel angle is between −1 and 1 through the activation function, where −1 represents a maximum left turn, and 1 represents a maximum right turn. Theoretically, the acceleration and deceleration should be controlled independently. Therefore, the sigmoid function is used to map the action directly to the range of [0, 1], where 0 indicates no operation, and 1 indicates maximum acceleration or deceleration. However, in practice, first, a human driver will not step on the gas and brake simultaneously in the normal operation of the vehicle. Second, if the acceleration and deceleration are decided independently, it may appear that the agent will decelerate while accelerating in order to obtain a lower resultant acceleration, which is not advised in terms of fuel economy and safety. Through the above analysis, we finally output the acceleration and deceleration control as a parameter through the tanh function, where −1 indicates maximum deceleration, and 1 indicates maximum acceleration. The critic-network combines the preprocessed data with the action of the actor-network output as the input, connects through the fully connected layer, uses the rectified linear unit as the activation function in the second hidden layer, and finally outputs the *Q* value. Table 1 and Table 2 present the structure of the actor-network and critic-network. The input to the actor-network is the number of features in the state space, whereas the input to the critic-network is the sum of the state features and actions. Both networks have two fully connected layers between the input and output layers, and the number of neurons in the first fully connected layer is 150, and the number of neurons in the second layer is 20. The output of the actor-network represents the steering wheel angle and the acceleration of the agent, whereas the output of the critic-network represents the corresponding *Q*-values.

### 3.4. Construction of Simulation Environment

**Figure 4:**
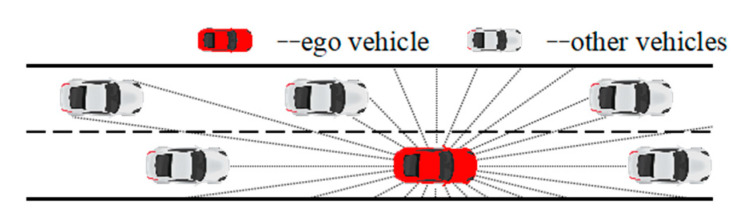
Visual result of simulation environment.

Without considering the influences of traffic signs, weather conditions, and other factors, a driver’s attention is mainly focused on the geometric information of the road and objects moving on the road in the process of lane change. The geometric information of the road mainly includes the curvature of the road and the location of the lane. The moving objects on the road mainly include vehicles moving in the motor vehicle lane (including people involved in traffic and non-motor vehicles at road intersections). Therefore, in the process of constructing the automated lane-changing environment, we consider the lane line, the lane boundary line, and the vehicles in each lane. The necessary conditions for the safe driving of autonomous vehicles are as follows: (1) The vehicle is driving in the lane of the road, (2) A safe distance is maintained from other vehicles. Without considering pedestrians, non-motor vehicles, and other traffic participants, the simulation environment includes several key elements such as the lane boundary, road centerline, and surrounding moving vehicles. Based on the above requirements, we construct a physical environment based on Python, use the Pyglet module to visualize the simulation environment, and obtain the real-time data of the detected lane and the surrounding vehicles through the lidar (manufacturer, city, state if in US, country). Figure 4 shows the visualization result of the simulation environment.

### 3.5. State Space Design

**Figure 5:**
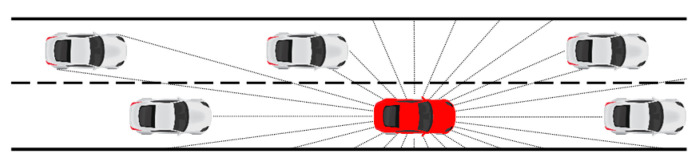
State space of automated lane change.

After a comprehensive consideration of all the aspects, we finally placed a simulated single-line lidar in the autonomous vehicle in simulation environment to detect and perceive the motion information of the surrounding vehicles and the road lines at both sides. An autonomous vehicle can judge its own relative position through perception to obtain the distance state information between itself and its surrounding vehicles and the lanes at both sides, thus better achieving a series of driving tasks such as lane keeping and automated lane change. Therefore, in this study, the state space of the MDP is defined as the distance state $D_{lane}$ of the lane detected by the lidar and the distance state $D_{vehicle}$ between the agent and the other vehicles around it. To fully perceive the motion information of the surrounding vehicles, as shown in Figure 5, the single-line lidar detection range is defined as [0°, 360°]. The range is divided into 60 equal parts (which can ensure that the vehicle within the detection range will always have at least one distance return value during the vehicle steering process), and the maximum lidar detection range is 50 m. The state space is defined as:

(6) $$State=\left\{D_{lane},D_{vehicle}\right\}$$

Among them, the state space contains 61 data values of the distance $D_{lane}$ between the lanes detected by lidar and the distance $D_{vehicle}$ from the surrounding vehicles.

### 3.6. Action Space Design

The action space is the sum of the actions that the agent can take in terms of the lane-changing behavior. In order to ensure the security, efficiency, and comfort in the lane-changing process, the steering wheel angle, throttle opening, and brake pedal pressure should be controlled. This will affect the front wheel angle, acceleration, and other motion parameters. In terms of the lateral control, considering that the input to the vehicle kinematics model is the front wheel steering angle, the steering system structure is simplified, and the steering system angle transmission ratio is assumed to be 17:1. The position and attitude adjustment of the agent in the environment is realized by controlling the steering wheel angle. Aiming at the DDPG algorithm, which can represent the continuous actions, we use the continuous action space as the control range of the steering wheel angle. The action space in the lateral aspect is defined as:

(7) $$Action=\left\{\theta_{w}\right\}$$

Automatically changing the lane of vehicles requires coordinating the relationship between the steering and the speed, in order to ensure that the task of lane change can be completed safely and quickly. In addition to controlling the steering wheel angle of the vehicle, the action space should also control and adjust the longitudinal speed of the vehicle, so the longitudinal action space is set to the normalized longitudinal acceleration a. Here, a∈[−1, 1], where a negative number indicates deceleration, zero indicates a constant speed, and a positive number indicates acceleration.

To sum up, the comprehensive action space considering the angle control of the lateral steering wheel and longitudinal speed control can be defined as:

(8) $$Actions=\left\{\theta_{w},a\right\}$$

### 3.7. Reward Function Design

The reward function is related to whether the agent can successfully complete the task in reinforcement learning and some performance requirements that need to be met on the basis of completing the task. Therefore, reasonably designing the reward function is the key to guide the agent to complete the lane change task and meet the performance requirements. The design of the reward function is mainly based on security, comfort, and efficiency:

1. Security:

Security is the most important evaluation factor in the process of automated lane change of intelligent vehicles. Security should be taken as the premise and the primary consideration in the entire lane change process, so as to avoid any traffic accidents and property loss. In this study, the design of the reward function for security is mainly considered from two aspects: avoiding collision between the agent and other vehicles and setting different expected lane-changing distances to represent different driving styles.

First, it is considered that the agent should avoid collisions with the surrounding vehicles and give the corresponding negative reward in the event of a collision. The specific design is as follows:

(9) $$R_{collision}=k_{c}\times C_{flag}$$

(10) $$C_{flag}=\{\begin{array}{l} 1,\;if\;collision\\ 0,\;else \end{array}$$

where $k_{c}$ is the reward weight coefficient of the collision between the agent and other vehicles, *k_c_* < 0, and $C_{flag}$ is the detection mark of the collision between the agent and other vehicles.

Subsequently, it should be considered that different driving styles can be characterized by setting different expected lane-changing distances from the vehicles ahead. The expected lane-changing distance is taken as the threshold, so that when the distance between the agent and the vehicle in front is less than the threshold, the penalty value will gradually increase with the increase in the desired lane-changing distance, thus improving the security. The specific design is as follows:

(11) $$R_{distance}=k_{t}\left|d-d_{des}\right|$$

where $k_{t}$ is the reward weight coefficient at the time of lane change, $d_{des}$ is the expected lane-changing distance between the agent and the vehicle in front, $k_{t}$ < 0. By setting different expected lane-changing distances, we can use the reward function to represent different driving styles.

Drivers have different driving styles while changing lanes, such as conservative and aggressive. A conservative driving style is when one’s vehicle is controlled to change lanes only when there is a large distance from the vehicle in front, while an aggressive driving style is when one’s vehicle is controlled to change lanes even when the distance from the vehicle in front is very small. As mentioned above, by setting $d_{des}=10\;m$, we can train the agent to change lanes as soon as possible when the distance from the vehicle in front is less than 10 m, thus characterizing a conservative driving style. By setting $d_{des}=0\;m$, the training agent starts to change lanes only when it is about to collide with the vehicle in front, thus characterizing an aggressive driving style.

To sum up, the reward function that considers the security of agents can be expressed as follows:

(12) $$R_{security}=R_{collision}+R_{distance}$$

2. Comfort:

When considering the ride comfort of drivers and passengers, the lateral and longitudinal aspects of autonomous vehicle movement should be fully considered. The angular value of the front wheel should not change too fast in the lateral motion control, so the angular velocity of the steering wheel is used as an index to evaluate the comfort. The angular speed of the steering wheel is the rate of change in the steering wheel angle. The lower the angular speed of the steering wheel, the smoother the lane changing process of the vehicle, and the passengers will experience better ride comfort. The comfort reward for lateral control can be expressed as follows:

(13) $$R_{comfort}=k_{w}\times \dot{\theta_{w}}$$

where $k_{w}$ is the reward weight coefficient of the steering wheel angular speed, $\dot{\theta_{w}}$ is the angular velocity of the agent steering wheel, $k_{w}$ < 0.

To avoid a significant change in the acceleration during the longitudinal motion control of autonomous vehicles, the jerk is used as an index to evaluate comfort. The jerk reflects the rate of change in the acceleration. The lower the jerk in the lane-change process, the smoother the acceleration process of the vehicle, thus ensuring better ride comfort. The reward function for considering agent comfort during vertical control is as follows:

(14) $$R_{comfort}^{\prime }=k_{a}\times \dot{a}$$

where $\dot{a}$ is the jerk of the agent, and $k_{a}$ is the jerk reward weight coefficient, $k_{a}<0$.

To sum up, the reward function considering agent comfort is:

(15) $$R_{comfort}^{\prime \prime }=k_{w}\times \dot{\theta_{w}}+k_{a}\times \dot{a}$$

3. Efficiency:

In the lane-changing process of intelligent vehicles, the efficiency of the entire lane change process should also be considered. This is to ensure that the agent can quickly change from the current lane to the target lane, while maintaining the necessary driving speed in the entire lane changing process, so as to avoid increasing the time required for changing lanes.

The reward function that enables the agent to quickly change from the current lane to the target lane is designed as follows:

(16) $$R_{efficiency}=k_{k}\left(\left|TL-y\right|+k_{1}\left(\frac{2-LN}{2}\right)\left(1-LN\right)\left|TL-y\right|\right)$$

(17) $$TL=\{\begin{array}{c} 120,\;if\;LN=1\\ 160,\;if\;LN=2\\ 140,\;else\;LN=0 \end{array}$$

where TL is the target lane, LN is the lane number, and the values of 0, 1, and 2 indicate that the current position of the agent is not in the driving area, in lane 1 (left), and in lane 2 (right), respectively. y is the lateral position coordinate of the agent, and $k_{k}$ and $k_{1}$ are constant terms, where $k_{k}<0$ and $k_{1}>0$. For Equation (15) and in combination with Figure 4, when the agent is in lane 1 or lane 2, it corresponds to LN=1 or LN=2, respectively, and $k_{1}\left(\frac{2-LN}{2}\right)\left(1-LN\right)\left|TL-y\right|$ in this formula is zero. In addition, the denominator of (2−LN)/2 is to ensure that the same reward is given in both cases (whether the agent leaves the lane from the left or from the right). When LN=0, i.e., after the agent leaves the lane and enters the non-driving area, $k_{1}\left(\frac{2-LN}{2}\right)\left(1-LN\right)\left|TL-y\right|$ is not zero but increases the additional overall negative reward value.

Considering that the speed of the agent in the entire lane change process cannot be too low, the allowable minimum speed $v_{min}$ is limited, and the corresponding reward function is designed as follows:

(18) $$R_{speed}=k_{l}\times L_{flag}$$

(19) $$L_{flag}=\{\begin{array}{c} 1,\;v<v_{min}\\ 0,\;v\geq v_{min} \end{array}$$

where $k_{l}$ is the weight coefficient of the agent speed limit reward, $k_{l}<0$, and $L_{flag}$ is the agent speed limit detection mark.

Therefore, the reward function considering the efficiency of the agent in the entire lane change process is as follows:

(20) $$R_{efficiency}^{\prime }=R_{efficiency}+R_{speed}$$

Based on the analysis and consideration of the security, comfort, and efficiency, the total reward function of the agent under the joint lateral and longitudinal control can be expressed as follows:

(21) $$R^{\prime }=R_{security}+R_{comfort}^{\prime \prime }+R_{efficiency}^{\prime }$$

## 4. Materials and Methods

### 4.1. Simulation Environment Settings

In this study, the simulation environment is based on the physical environment constructed using Python, and the scenarios are visualized through Pyglet. In the simulation environment, we use the mathematical method of the intersection of two vectors in a 2D plane to calculate the Euclidean distance of the agent from the lane and other vehicles in all directions. The bicycle model in vehicle dynamics is used to describe the basic motion law of the agent. Moreover, the collision detection between the agent and other vehicles is added. Finally, we train the agent to change lanes independently in a one-way two-lane road, where the length of the simulated road is 100 m, the width of the lane is 3.75 m, the length of the car body is 5 m, and the width is 2 m. Table 3 presents the software and hardware environments that the agents rely on in the training process.

### 4.2. Parameters Setting in Simplified Traffic Scene

To verify the difference between the lane-changing process and the lane-changing time for agents with different driving styles (conservative and aggressive), we set up a simplified traffic scene, i.e., the influence of rear vehicles is not considered for the time being. We only set a front vehicle on the target lane and the current lane to form a typical scene. First, the model is trained to ensure that the agent can perform a series of driving tasks such as lane change, and then explore the impact of different driving styles on the lane-change process and whether comfort is ensured.

Based on the task requirements presented in this section, the traffic scene of the training model is simplified to some extent, and only the influence of the vehicle in front of the agent is considered. Table 4 lists some of the specific parameters of the DDPG algorithm used in the training process, such as the learning rate, the maximum number of episodes, the maximum number of steps, and the weight coefficient of the reward function. The entire driving task can be divided into the following three parts:

1. When the lane-change condition is not reached, the agent remains in the current lane;
2. After the lane-change condition is reached, the agent changes the lane automatically;
3. After the lane change is completed, the agent remains in the new lane.

Based on the requirements of the experimental task and the design of the experimental scheme, the model training was carried out for a total of 600 episodes, and 100 steps were set for each episode of training. In the one-way two-lane road, the initial speed of the agent is 30 km/h, the speed of the front is 20 km/h, and the speed of the left front is 28 km/h. At the initial position, the distance between the agent and the front vehicle in the current lane is 20 m.

### 4.3. Parameters Setting in Complex Traffic Scene

To simulate the real driving scene and traffic flow as much as possible, we performed a study in the case where there are vehicles in the front and rear of the surrounding target lane and the current lane. Agents representing different driving styles complete a series of driving tasks, such as lane change and adaptive speed adjustment, through joint lateral and longitudinal control, so that the agent can adapt to complex dynamic traffic flow and compare the differences between different driving styles.

To ensure that the agent can cope with different traffic flow conditions in the training process, a dynamic traffic scene is designed in the experimental scheme. In the training phase, the simulation environment will automatically adjust the position of the vehicles in the front and rear of the left lane. At the same time, as done previously, the aggressive and conservative driving styles are set to distinguish and compare the impact of driving styles on the entire lane-changing process. Table 5 lists the training parameters used in the training process (the remaining parameters are detailed in Table 4). The experimental task consists of two parts:

1. Complete the task of changing lanes in the dynamic traffic scene;
2. After the lane change is completed, adjust the speed to adapt to the current lane traffic flow.

Based on the requirements of the experimental task and the experimental scheme, the agents representing the two driving styles will carry out 900 episodes of training, with 60 steps per episode. In the one-way two-lane road, the initial speed of the agent is 40 km/h, the speed of the front vehicle is 20 km/h, the speed of the rear vehicle is 22 km/h, the speed of the left front vehicle is 28 km/h, and the speed of the left rear vehicle is 25 km/h. At the initial position, the distance between the agent and the front vehicle is 13 m, and the distance between the agent and the rear vehicle is 8 m.

## 5. Results

### 5.1. Training Results in Simplified Traffic Scenes

**Figure 6:**
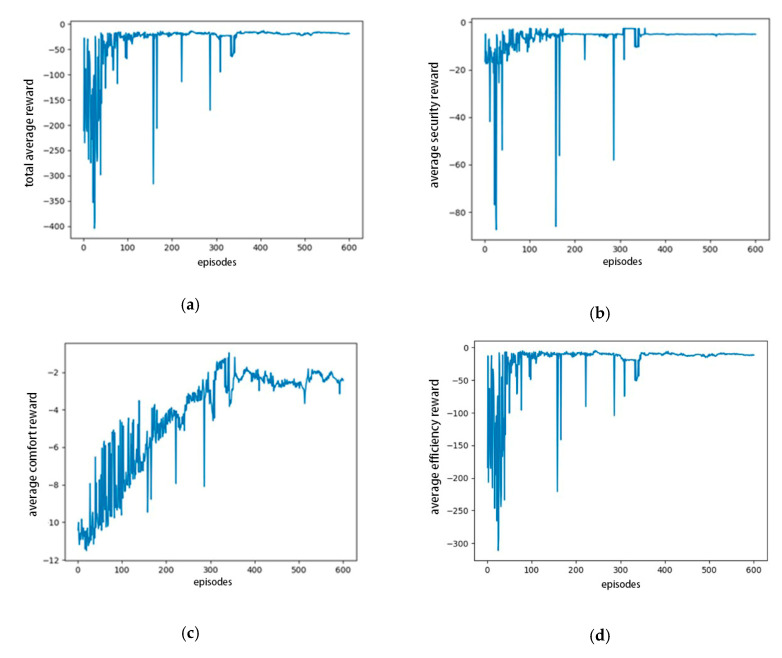
Rewards of model training: (**a**) total average reward, (**b**) average security reward, (**c**) average comfort reward, (**d**) average efficiency reward.

Figure 6 shows the average of all types of reward images of the agent during the training process, including the total reward, security reward, comfort reward, and efficiency reward. Figure 6a shows that the reward exhibits a downward trend at the beginning of training because the agent is constantly balancing the relationship between exploration and utilization. With continuous training, the average reward gradually converges, indicating that the agent can learn the lane-changing policy to maximize the reward function. Figure 6b–d shows the average rewards of the security, comfort, and efficiency in the training process, respectively, all of which increase and then gradually converge. This indicates that under the design of the reward function considering security, comfort, and efficiency, the agent can ensure that the lane is changed safely without any collision. Moreover, the comfort is significantly improved, and the lane-change task is completed efficiently.

### 5.2. Verification Results in Simplified Traffic Scenes

This section presents the differences in the lane-changing behaviors of agents under the control of different driving styles, and the lane-changing process tracks of agents with different driving styles are compared by setting an identical scene to determine the differences. Subsequently, a single-episode reward of the agent with different initial speeds in the lane-change process under the conservative driving style is analyzed, and the changes in the angular velocity of the steering wheel with and without considering comfort are compared to verify whether the comfort of the lane-change process is ensured.

**Figure 7:**
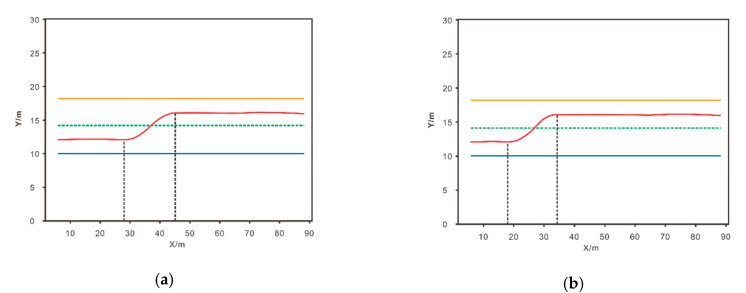
Tracks of lane change with different driving styles in the same scene: (**a**) aggressive driving style, (**b**) conservative driving style.

Figure 7 shows the track position graphs of the agent completing the lane-change process under different driving styles. Figure 7a shows that under the aggressive driving style, the agent begins to change lanes at 28 m and completes the lane change at 45 m. Under the conservative driving style shown in Figure 7b, the agent begins to change lanes at 18 m and completes the lane change at 34 m. A simple calculation shows that the lane-changing process of the agent runs for 17 m in the x direction in the aggressive driving style and 16 m in the x direction in the conservative driving style. Under the condition that the speed of the agent is the same, the time of lane change is the same. However, under the same scene and the same speed, the lane-changing position of the agent with the aggressive driving style is 10 m later than that of the agent with the conservative driving style under the same conditions, and the lane-changing time is even later.

**Figure 8:**
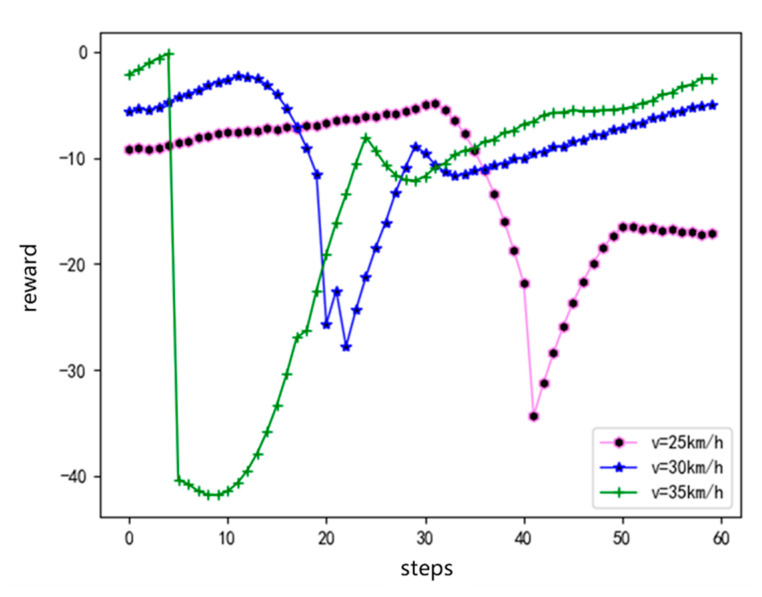
Single-episode rewards at different speeds.

**Figure 9:**
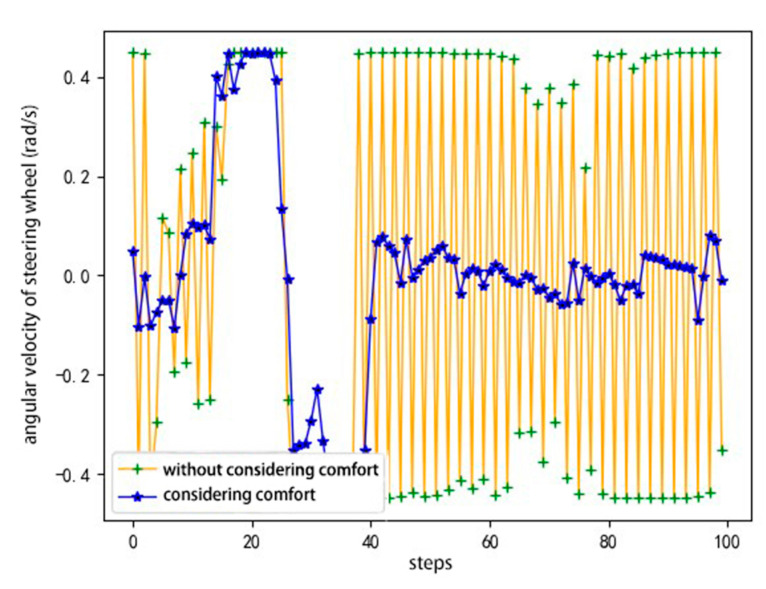
Angular velocity of steering wheel.

In view of the lane-change process of the agent in the conservative driving style, the following are compared and verified at different speeds. As shown in Figure 8, the agents carry out lane-changing experiments at speeds of 25, 30, and 35 km/h. As shown, the reward of the single episode of the agents at different speeds exhibits an increasing trend. Figure 9 shows the verification results of the angular velocity change of the steering wheel with and without considering comfort reward. The purpose of considering the comfort reward is to decrease the angular velocity of the steering wheel. Through the comparison curve with and without considering comfort in the figure, it can be clearly seen that the angular velocity of the steering wheel is lower, and the overall curve tends to 0 when considering the comfort reward, indicating that the comfort in the lane-change process has been effectively ensured. The verification results show that the agent can complete the driving task and meet the requirements of security, comfort, and efficiency using the model based on DDPG training and the designed reward function at different speeds.

### 5.3. Training Results in Complex Traffic Scenes

**Figure 10:**
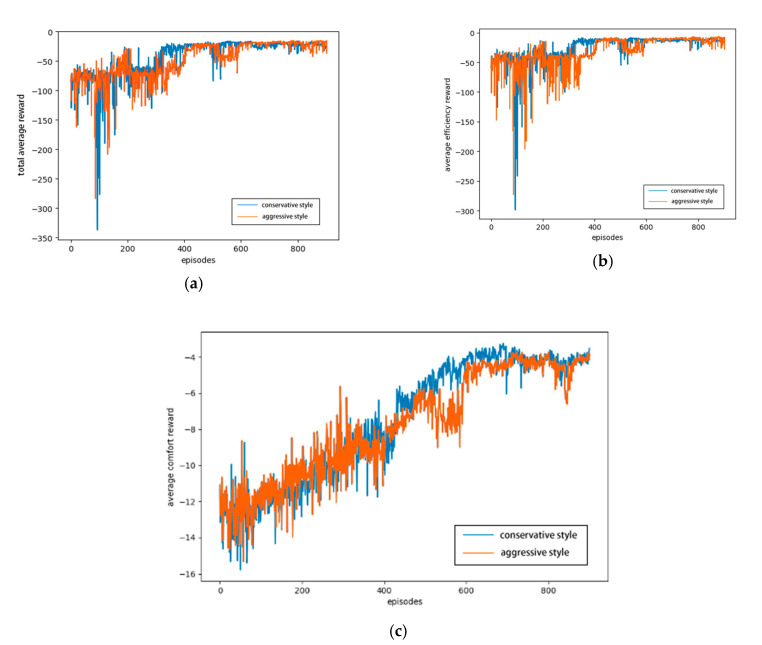
Rewards of different driving styles: (**a**) total average reward, (**b**) average efficiency reward, and (**c**) average comfort reward.

Figure 10 shows the average rewards of the agents with two different driving styles under the joint lateral and longitudinal control, including the total average reward, average comfort reward, and average efficiency reward.

Figure 10a shows that the total average rewards of the two driving styles constantly fluctuate at the beginning of the training. However, with the continuous iteration of the number of training episodes, the average rewards of the conservative and aggressive driving styles tend to converge after 600 episodes, proving that a series of driving tasks, such as automated lane change and lane keeping, can be achieved with these driving styles in the later stage. The agent can learn the lane-changing policy to maximize the value of the reward function. On the other hand, the reward function curve of the agent representing the conservative driving style is evidently more convergent, smoother, and faster than the aggressive one in the entire process and later stage, which indicates that the agent with a conservative driving style is more effective in completing driving tasks. In the same lane-changing scene, due to the different lane-changing times of the two driving styles, there are some differences in the reward images of efficiency and comfort. For example, with the conservative driving style, the agent can reach the target lane as soon as possible, so it is more efficient because the lane-changing time is earlier. Figure 10b shows that the overall reward function curve of the conservative driving style is smoother and more convergent than that of the aggressive driving style, indicating that the lane-changing process of the agent of the conservative driving style is more efficient. From the average comfort reward function shown in Figure 10c, the comfort of the two driving styles is constantly improved and optimized, whereas the reward function curve of the conservative driving style is smoother and convergent. Regardless of the driving style, Figure 10a–c shows a growing trend and gradual convergence, indicating that under the reward function of security, comfort, and efficiency, the agent can ensure a safe lane change without any collision. Moreover, the comfort is significantly improved, and the lane change task can be completed efficiently.

**Figure 11:**
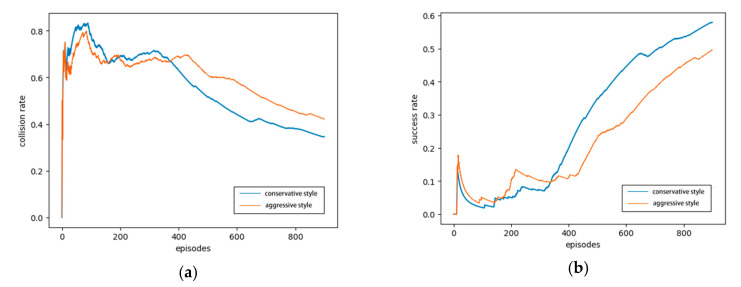
Success and collision rates of different driving styles: (**a**) collision rate, (**b**) success rate.

Figure 11a shows that the collision rate of the agent with a conservative driving style decreases faster.

For example, at the 600th episode, the collision rate of the conservative agent is approximately 15% lower than that of the aggressive agent, and in the final result, the collision rate of the conservative agent is approximately 10% lower than that of the aggressive agent. Figure 11b shows that the lane-changing success rate of the conservative agent increases faster, and the upward trend is more evident. The lane-changing success rate of the aggressive agent is more volatile at the beginning, and in the final result, the lane-changing success rate of the conservative agent is approximately 10% higher than that of the aggressive agent, indicating that the conservative agent can better complete the automated lane change and other driving tasks. As shown in Figure 11, the lane-changing success rates of the two styles continue to increase with the number of iterations, whereas the collision rate decreases with the iteration. Both of the curves show that the trained agent ensures security in the lane-change process, while the security and success rate of the conservative driving style are higher.

### 5.4. Verification Results in Complex Traffic Scenes

In this study, we verified the lane-changing behavior of the agent in complex dynamic traffic flow scenarios from the following aspects: (1) Single-episode reward of agents at different initial speeds, (2) Single-episode reward of agents under different average speeds in the road section, (3) The influence of comfort reward on the angular velocity of the steering wheel and jerk.

**Figure 12:**
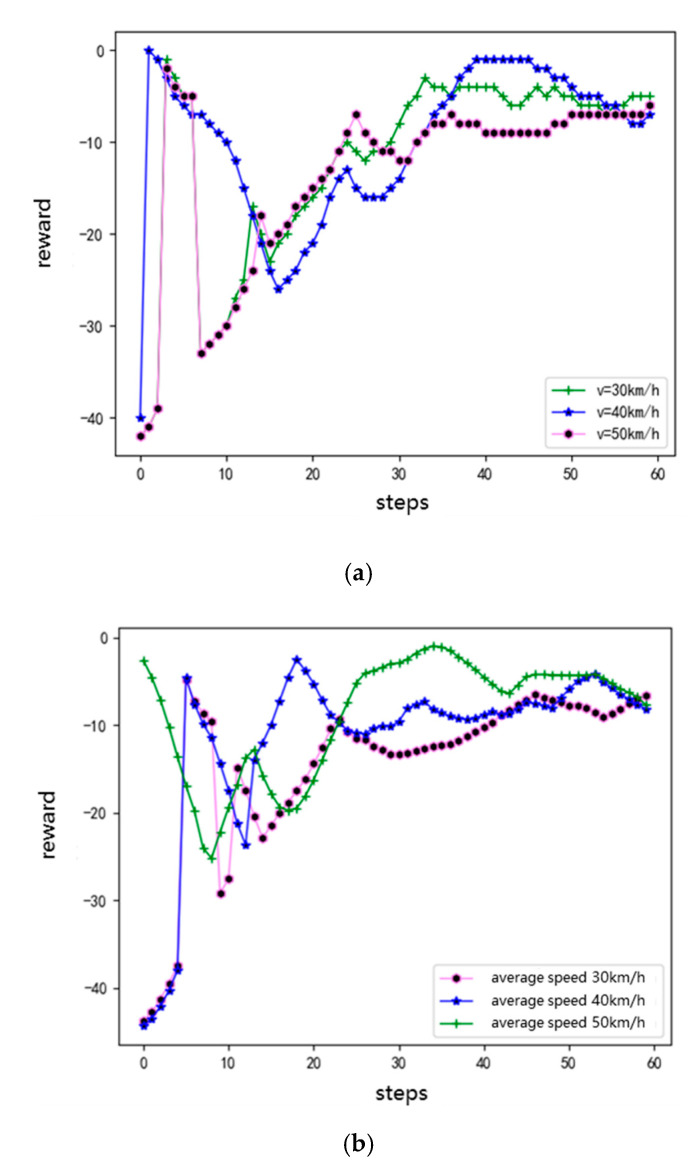
Single-episode rewards of agents in different scenarios: (**a**) different initial speeds, and (**b**) different average speeds in the road section.

Figure 12a shows a comparison of the rewards of the agents with initial speeds of 30, 40, and 50 km/h. Figure 12b shows a comparison of the rewards of the surrounding road traffic running at average speeds of 30, 40, and 50 km/h, respectively. Clearly, the reward curves of the agents show an increasing trend under different initial speeds or different average speeds in the road section. The overall trend in the reward curve is the greatest when the average speed of the traffic flow in the surrounding section is 50 km/h. Moreover, the figure shows that the greater the average speed of the traffic flow in a certain range, the greater the reward for the agent.

**Figure 13:**
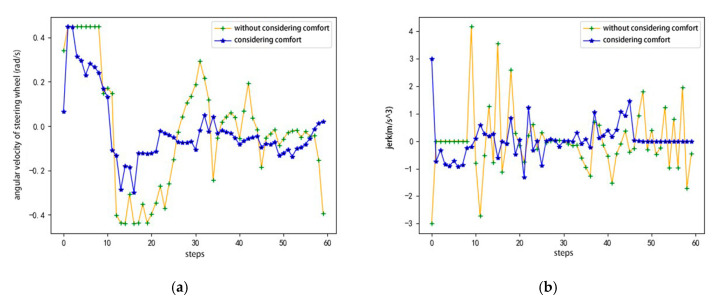
Comparative results with and without considering comfort reward: (**a**) angular velocity of steering wheel, and (**b**) jerk.

Figure 13a,b shows the comparative verification results of the angular velocity and jerk of the agent, respectively, with and without considering the comfort reward.

The purpose of considering the comfort reward is to minimize the jerk and angular velocity of the steering wheel. By comparing the curves with and without the comfort reward, we see that the agent with the comfort reward outperforms the agent without the comfort reward in terms of the angular velocity of the steering wheel and jerk overall. The above verification results show that the agent has good robustness under different speeds and different average speeds in the road section, and the model based on DDPG training and the designed reward function can meet the requirements of security, comfort, and efficiency.

## 6. Conclusions

In this study, we developed an end-to-end reinforcement learning framework for the automated lane-change control of intelligent vehicles based on lidar detection data. The simulation environment was constructed using Python, and the simulation environment was visualized in Pyglet. In the simulation environment, a lidar was used to detect the lane boundary and the distance information of the surrounding vehicles, which together constitute the driving area for the lane change and are directly used as the original data input in the state space. A deep deterministic policy gradient (DDPG) algorithm was adopted. The actor–critic network structure of the DDPG algorithm uses a fully connected neural network to determine the output of the continuous actions. The state space uses the state distance information from the agent to the surrounding vehicle and lane boundary obtained by the lidar in the simulation environment as the training data. The continuous steering wheel angle and longitudinal acceleration were used as the action output in the action space. In terms of the reward function, avoiding collision and setting different expected lane-changing distances that represent different driving styles were considered for security, whereas the angular velocity of the steering wheel and jerk were considered for comfort. The minimum speed limit required for changing lanes and the control of the agent for a quick lane change were considered for efficiency. Two types of agents representing different driving styles were designed, and their differences in terms of the lane-changing process and lane-changing time were compared in simplified traffic scenarios. In complex traffic scenarios, an automated lane-changing training was carried out through joint lateral and longitudinal control, and the differences were obtained by comparing and analyzing the reward function, collision rate, and success rate under the two styles. The final training and verification results showed that the proposed method can enable the agent to change a lane safely, smoothly, and efficiently in a one-way two-lane road.

Some areas need to be explored further in depth. The verification part of this paper was carried out in a simulation environment, without any experiments in a real environment. Considering the security for real vehicle verification, follow-up tasks can be verified on a model car. The process of changing lanes involves the cooperative lane-changing problem of multi-vehicle game theory on current and target lanes. In follow-up studies, a multi-agent can be introduced in this regard.

## Figures and Tables

**Table 1:** Actor-network structure.

Name	Size	Annotation
Input layer	n	State space
Fully connected layer 1	150 × 1	Number of neurons in layer 1
Fully connected layer 2	20 × 1	Number of neurons in layer 2
Output layer	2 × 1	$\left\{\theta_{w},a\right\}$

**Table 2:** Critic-network structure.

Name	Size	Annotation
Input layer	n + 2	State space + action
Fully connected layer 1	150 × 1	Number of neurons in layer 1
Fully connected layer 2	20 × 1	Number of neurons in layer 2
Output layer	1 × 1	*Q*-values

**Table 3:** Software and hardware environment.

Hardware and Software	Model/Version
CPU	Intel Core i7-4770
RAM	16.0 GB
Operating system	Windows Server 2019 64-bits
Python	3.7
Deep learning framework	TensorFlow-1.14
Visualization tool	Pyglet-1.2.4

**Table 4:** Parameters of model training.

Parameter Name	Parameter Value	Parameter Name	Parameter Value
MAX_EPISODES	600	MEMORY_CAPACITY	2000
MAX_EP_STEPS	100	BATCH_SIZE	64
Actor-network initial learning rate	0.001	Critic-network initial learning rate	0.001
γ	0.9	$k_{k}$	−1
$k_{t}$	−0.1	$k_{1}$	0.1
$k_{w}$	−0.4	$k_{c}$	−200

**Table 5:** Lateral and longitudinal training parameters.

Parameter Name	Parameter Value	Parameter Name	Parameter Value
MAX_EPISODES	900	MEMORY_CAPACITY	2000
MAX_EP_STEPS	60	BATCH_SIZE	64
Actor-network initial learning rate	0.001	Critic-network initial learning rate	0.001
γ	0.9	$k_{a}$	−0.4
$k_{l}$	−10	$v_{min}$	4.17 (m/s)

## Abbreviation

DDPG	Deep Deterministic Policy Gradient
MDP	Markov Decision Process
DQN	Deep Q Network
TORCS	The Open Racing Car Simulator
CAN	Controller Area Network
GPS	Global Positioning System
A3C	Asynchronous Advantage Actor–Critic
WRC6	World Rally Championship 6
DPG	Deterministic Policy Gradient
